# Potential of Fe-Mn-Al-Ni Shape Memory Alloys for Internal Prestressing of Ultra-High Performance Concrete

**DOI:** 10.3390/ma16103816

**Published:** 2023-05-18

**Authors:** Maximilian Schleiting, Alexander Wetzel, André Bauer, Johanna-Maria Frenck, Thomas Niendorf, Bernhard Middendorf

**Affiliations:** 1 Department of Structural Materials and Construction Chemistry, University of Kassel, 34125 Kassel, Germany; alexander.wetzel@uni-kassel.de (A.W.); middendorf@uni-kassel.de (B.M.); 2 Institute of Materials Engineering, University of Kassel, 34125 Kassel, Germany; bauer@uni-kassel.de (A.B.); j.frenck@uni-kassel.de (J.-M.F.); niendorf@uni-kassel.de (T.N.)

**Keywords:** high-performance concrete, prestressing, shape memory alloys, bond strength

## Abstract

Prestressing of concrete is a commonly used technique in civil engineering to achieve long spans, reduced structural thicknesses, and resource savings. However, in terms of application, complex tensioning devices are necessary, and prestress losses due to shrinkage and creep of the concrete are unfavourable in terms of sustainability. In this work, a prestressing method using novel Fe-Mn-Al-Ni shape memory alloy rebars as a tensioning system in UHPC is investigated. A generated stress of about 130 MPa was measured for the shape memory alloy rebars. For the application in UHPC, the rebars are prestrained prior to the manufacturing process of the concrete samples. After sufficient hardening of the concrete, the specimens are heated inside an oven to activate the shape memory effect and, thus, to introduce the prestress into the surrounding UHPC. It is clearly shown that an improvement in maximum flexural strength and rigidity is achieved due to the thermal activation of the shape memory alloy rebars compared to non-activated rebars. Future research will have to focus on the design of the shape memory alloy rebars in relation to construction applications and the investigation of the long-term performance of the prestressing system.

## 1. Introduction

Ultra-High-Performance Concrete (UHPC) is characterised by a compressive strength above 150 MPa and high durability. These properties are based on a high packing density, a low water/binder ratio < 0.25, and the use of reactive fillers [1].

In contrast to its high compressive strength, the tensile and flexural strength of UHPC are comparatively low. Furthermore, the failure behaviour of UHPC is brittle and abrupt due to its high packing density [1,2,3]. To improve these characteristics, reinforcement in the form of fibres or rebars (or a combination of both) is added to the concrete [4,5,6]. Fibre reinforcement is used to introduce a more ductile failure behaviour, while a rebar reinforcement strongly improves the tensile and flexural strength of the concrete [7,8,9,10,11,12]. However, buildings with reinforced concrete must adhere to different regulations, e.g., the DIN Norm in Germany. These standards control the manufacturing of building components to ensure sufficient mechanical properties for the use case. Compliance with these regulations sometimes counteracts the environmental approach of using CO_2_-friendly building materials and, therefore, is not favourable in terms of life cycle assessment. The possibility of manufacturing material-saving components with sufficient mechanical properties is a task that has been examined for a long time now.

Current research in the fields of UHPC addresses these problems in different manners. The use of waste and recycled materials reduces the CO_2_ output of UHPC while maintaining sufficient mechanical properties [13,14,15]. Another approach is the optimisation of the mechanical properties by combination of different materials and using novel methods for applications [16,17]. One of these approaches is the use of prestressed high-strength steel rebars for reinforcement in concrete [10]. These rebars introduce a compressive stress into the concrete that counteracts the achieved tensile stresses in the application. This technique has been known for quite a long time. P.H. Jackson registered a patent for prestressed floor plates in 1886, and the first prestressed bridge was built in 1938 in Germany [18]. Today, prestressed concrete has reached nearly all parts of constructive engineering. Most prestressed concrete is used in bridge and building construction [10]. Prestressed concrete allows an increase in the spans of reinforced concrete beams, as well as reducing material components due to the enhanced mechanical properties.

**Figure 1:**
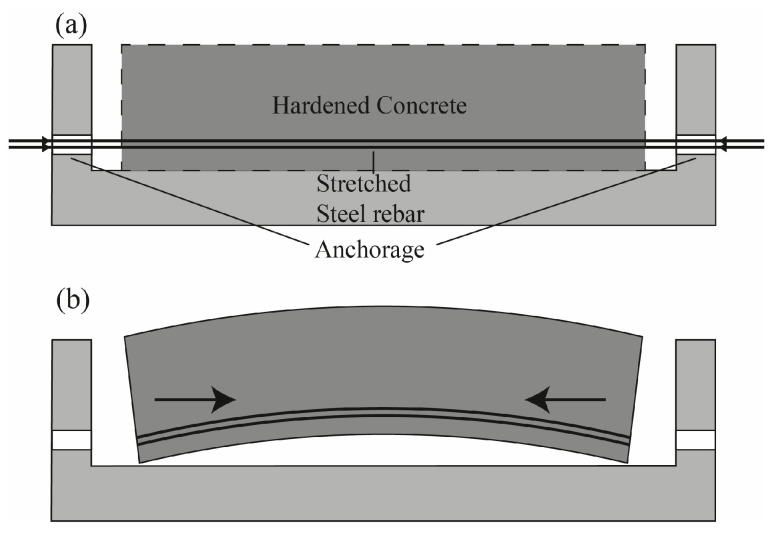
Schematic presentation of the prestressing principle. (**a**) The prestrained steel rebars are still anchored, and the surrounding concrete hardens to ensure a sufficient bonding between rebar and concrete matrix. (**b**) The steel rebars are detached from the anchoring device and, therefore, try to contract. The surrounding concrete prevents this contraction, and, thus, a stress is introduced into the concrete.

These advantages are based on a system that counteracts the resulting stresses in the application of a component. Therefore, steel rebars are placed in the area where the component will receive the highest tensile stresses. Subsequently, the rebars are elastically pre-tensioned with hydraulic devices and fixed externally on the concrete formwork while the concrete is poured around it. In the case of a bond-free prestress application, cladding tubes are used, and the concrete is prestressed due to an external anchorage [10]. After sufficient hardening of the concrete, the rebars are detached from the anchorage device and, therefore, try to contract due to their elasticity. The bonding with the surrounding concrete and the anchorage prevent this contraction and transfer the resulting stresses into the concrete (Figure 1).

The prestress counteracts the appearing stresses due to loads in the application. This increases the serviceability and, thus, increases the life cycle of the component. These advantages are mainly based on minimizing crack formation, less deformation of the component, and less susceptibility to vibration and fatigue [10].

In contrast to the named advantages, prestressed concrete also has some disadvantages. The main disadvantage is that the manufacturing process is very time-intensive and requires a complex setup to enable the prestraining of the rebars. Furthermore, shrinkage and creep of the concrete as well as relaxation of the prestressing steel can lead to a decrease in the prestress level [10]. Stress corrosion cracking is also a problem, as constantly stressed steel is much more susceptible to corrosion than regular steel [19]. Therefore, prestressed structures need to be monitored over time [10,20,21].

Recent research has shown the feasibility of using shape memory alloys (SMAs) as prestressing elements in concrete [22,23,24,25,26,27]. SMAs have the ability to return to their original imprinted shape after large deformations. Depending on the inherent properties of the SMA, the shape recovery is initiated upon removal of applied stress or upon heating of the SMA. The according shape memory phenomena are called superelasticity (SE) and the shape memory effect (SME) [28]. These unique properties are based on a reversible solid-state phase transformation between a martensitic low-temperature phase and an austenitic high-temperature phase and vice versa [29]. The SME was discovered in the late 1940s by Kurdjumov and Khandros [30], but the commercial breakthrough of SMAs was achieved with the development of the Ni-Ti alloy published in 1963 by Buehler et al. [31]. Ni-Ti is still the most commonly used SMA; however, its high costs resulting from expensive alloying elements and its challenging machining process [32] impede extended use, especially for material-intensive applications. With regard to applications in civil engineering structures, low-cost iron-based shape memory alloys have recently been the focus of research [33,34,35,36,37,38].

To utilize SMAs for prestressing applications in UHPC, the thermally induced SME is used. The procedure can be divided into three steps. The first one is prestraining of the SMA tendon. After subsequent unloading, the second step is the application to the construction element that has to be prestressed. Referring to the example of the prestressed concrete beam in Figure 1, the SMA rebar is placed into the mould in the area where the prestress is needed in the application. Finally, after pouring and sufficient hardening of the concrete, the activation of the SMA rebar is carried out. Therefore, the complete concrete element, including the SMA rebar, is heated up above the alloy-specific activation temperature. Due to the thermally induced phase transformation, the SMA rebar aims to reshape itself into its original imprinted (non-prestrained) shape. As it is bonded with the surrounding concrete, no strain recovery is possible and, therefore, a compression stress is imposed on the concrete beam. The thermal activation can be accomplished by convective heating with a heat source, conductive heating with electric current, or inductive heating.

Compared to conventional prestressing steels, the use of SMAs has several advantages. First, no anchorages and no hydraulic tensioning devices are necessary for applying prestresses to the concrete, which significantly simplifies the manufacturing process. Furthermore, this offers additional design opportunities, as the SMA rebars can also be placed in a curved geometry into the concrete, which is not possible with the conventional prestressing steel, if a prestressing in a fixed bond is envisaged.

The Fe-Mn-Al-Ni SMA used in this work is quite new. Vollmer et al. [35] demonstrated the prestressing potential of Fe-Mn-Al-Ni for the first time in 2021. The functional properties of Fe-Mn-Al-Ni are strongly related to nano-sized precipitates being present after solution annealing and subsequent quenching. The transformation temperatures are strongly related to the size of these precipitates. By applying an aging heat treatment at temperatures around 200 °C, the size of the precipitates increases, leading to a shift in transformation temperatures to lower values. Therefore, the alloy can show the SME in the initial state and the SE after aging. The modification of functional properties can be very beneficial for prestressing applications. The activation of the SMA prestressing element in the temperature regime causing precipitate growth enables multi-stage, tailorable prestress levels. Moreover, the change in functional properties from SME to SE can reduce prestress losses. The prestressing level can be tailored by the maximum heating temperature. Moreover, lower heating temperatures can be compensated by longer dwell times of heating. The maximum prestress level shown by Vollmer et al. for Fe-Mn-Al-Ni was about 485 MPa and is, therefore, slightly lower than but in the general range of regular prestressing steels [39]. However, it should be mentioned that the functional properties in Fe-Mn-Al-Ni are strongly influenced by the microstructure present, which requires precise temperature control during heat treatment and quenching.

This study investigates the use of the Fe-Mn-Al-Ni SMA as a prestress reinforcement in UHPC. The high strengths of the UHPC as well as the high amount of binder result in an enhanced bonding between rebar and cementitious matrix [40] and, therefore, are most suitable for the prestress application. The aim of this study is to generate a proof of concept for the general suitability of the Fe-Mn-Al-Ni SMA as a prestress element for UHPC. To have a general comparison to reference material, additional investigations with steel rebars were also carried out. To quantify the bond strength, rebar pullout tests out of a UHPC were conducted. The influence of the activation of the SMA was determined by flexural bending tests of concrete prisms reinforced with SMA rebars as well as steel rebars with different diameters and in different setups.

## 2. Materials and Methods

### 2.1. Ultra-High-Performance Concrete (UHPC)

The UHPC mixture “M3Q” used in this work was well-known from prior study within the SPP 1182 framework funded by the German Research Foundation (DFG) [1,41]. Table 1 shows the materials and their total amounts in relation to a volume of 1 m^3^ as well as their relative amounts. The water–binder ratio of the mixture is 0.21, and the superplasticizer content is 1.1% by weight of cement (bwoc). Fibre reinforcement was not used in this study to exclude bond-modifying factors, as the focus is on the rebars’ chemical composition and surface and their influences on the bond strength. Furthermore, fibre reinforcement could lead to an overly strong bond between rebar and the cementitious UHPC matrix [4], and extensive tearing of the rebars could occur.

Intensive concrete mixers (Eirich) were used. In the case of the samples for rebar pullout, a 30 L mixer was used, and in the case of the flexural bending prisms, a 5 L mixer was used. In total, the mixing process lasts 10 min. Prior to the addition of water and superplasticiser, the raw materials were homogenised for one minute. The water and superplasticizer mixture were added slowly during a period of 1/2 min, while the mixing speed was increased. In a two-minute break after a total mixing time of three minutes, residual dry components were removed from the mixing tool and container. Returning to the initial slower mixing speed, the material was stabilised for another five minutes.

For rebar pullout tests, moulds with dimensions of 100 mm × 100 mm × 100 mm were used. The rebars were dipped into the concrete from above and fixed to ensure a bonding length of 25 mm ± 2 mm. The flexural bending tests were carried out on prisms with dimensions of 40 mm × 40 mm × 160 mm. Therefore, holding devices for the rebars were 3D-printed to ensure a defined location of the reinforcement in the moulds.

**Figure 2:**
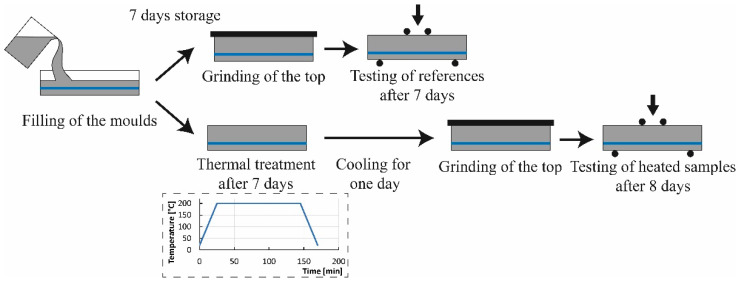
Manufacturing, thermal treatment, and testing procedure of the samples. The samples were stored at standard condition (65% rel. humidity and 20 °C) for seven days after mixing procedure. The samples that did not receive any thermal treatment were tested after seven days. The samples that were heated inside the oven received the thermal treatment seven days after mixing procedure shown in the dotted diagram. The dotted diagram shows the heating curve of the oven for the thermal treatment of the samples. The oven was heated to a temperature of 200 °C with a heating rate of 7 °C/min. After reaching 200 °C, the temperature was held for two hours to ensure a complete heating of the sample, including the SMA rebars. Afterwards, the specimens were cooled down to room temperature with the same rate (7 °C/min). The heated samples were then tested eight days after mixing procedure.

During filling, the moulds were placed on a vibrating table for 120 s at a frequency of 50 Hz to compact the concrete (Figure 2). In the case of the rebar pullout tests, the rebars were dipped into the concrete after 60 s of vibrating. All samples were demoulded after 24 h and stored at a temperature of 20 ± 2 °C and a relative humidity of 65 ± 5% before testing after 7 days.

To activate the SMA inside the samples and to generate the SMA and steel references without prestraining, these lab samples were heated inside an oven. The specimens were put into the oven at room temperature (20 ± 2 °C) and were then heated at a heating rate of 7 °C/min to 200 °C. This temperature was held for 2 h, and the samples were cooled at the same cooling rate to room temperature afterwards (Dotted diagram in Figure 2). This activation procedure was carried out after 7 days to ensure the same hydration of all samples. The testing for these samples was carried out after 8 days to ensure sufficient cooling of the samples and testing at standard condition. As the heating procedure was carried out after 7 days, the hydration difference after 8 days is assumed to be neglectable, and, thus, the results for those specimens are referred to as 7-day strength. To quantify the influence of the elevated temperatures on the non-reinforced concrete, the same thermal treatment was additionally applied to plain UHPC. For all samples, the tops of the prisms were grinded before testing to ensure a smooth surface, as the samples had to be tested in casting direction.

### 2.2. Fe-Mn-Al-Ni SMA and Steel Rebars

In this study, two different materials, regular reinforcement steel and Fe-Mn-Al-Ni SMA, are investigated regarding bonding behaviour and influence on flexural bending strength of reinforced UHPC. As the topographic microstructure as well as the internal metallic microstructure of the SMA rebars are important for their functionality and effectivity, these were also examined prior to the other tests.

To integrate the functionality into the SMA, the material has to be annealed to introduce the required microstructure. This was done at the Institute of Metal Forming of the Technische Universität Bergakademie Freiberg. The rebars themselves were obtained by a commercial manufacturer.

Annealing was done by heating the rebars up to 1250 °C and holding the temperature for 30 min. Due to the size of the rebars, annealing in a protective gas atmosphere was not possible. Therefore, the rebars were wrapped in steel foil together with Mn pellets to avoid a loss of Mn during heat treatment. Afterwards, the rebars were quenched in 80 °C warm water. By this process, rebars with diameter of 3 mm and rebars with diameter of 5 mm were produced for this work. To introduce a prestress into the UHPC, the SMA rebars had to be prestrained. Therefore, the rebars were prestrained in a universal testing machine up to 2%.

### 2.3. Methods

#### 2.3.1. Characterisation of the Rebar Materials

The chemical composition of the material was measured by energy-dispersive X-ray (EDX) spectroscopy, and the tensile strengths of the Fe-Mn-Al-Ni SMA as well as the steel rebars were determined with a tensile test upon rupture of the rebars. Before investigating the material behaviour in the composite, the functional properties of the SMA rebars were first investigated separately to quantify the maximum expected prestress level for the material batch. Furthermore, this approach should allow comparison with the results of Vollmer et al. [35]. The mechanical tests were carried out using a servo-hydraulic testing machine equipped with a 64 kN load cell. The SMA rebar was prestrained up to 2.3% and subsequently unloaded up to a stress level of 150 MPa. This stress level was chosen to avoid buckling of the rebars in the heating process. Finally, a heating–cooling cycle up to 250 °C with a dwell time of 30 min at activation temperature and heating–cooling rates of 0.5 K s^−1^ was carried out. The elongation was kept constant during the heating–cooling cycle. Temperatures were measured using a thermocouple type K attached directly to the specimen surface and controlled by convective heating with two hot-air guns.

For the microstructural investigations of the Fe-Mn-Al-Ni rebars, 30 mm long pieces of randomly chosen specimen were cut out of the rebars. Afterwards, they were fixed within epoxy resin. After drying and hardening, the specimens were grinded in several steps with decreasing grain sizes of the grinding material. Subsequently, the samples were polished. To visualise the microstructure of the SMA, the specimens were etched with a Nital mixture (2% concentrated nitric acid, 98% ethanol) for a few seconds and cleaned with distilled water directly afterwards. This was carried out for regular SMA rebars as well as for 2% prestrained SMA rebars. The internal microstructure was recorded with a digital light microscope (Keyence VHX 7000).

Regarding rebar pullout, the surface of the materials has a significant impact on the bonding between metal and UHPC. Both rebar types are considered to be smooth, as the rebars are not ripped or modified in any way after manufacturing. However, investigations of the surface using an environmental scanning electron microscope (ESEM) revealed distinct differences between steel and SMA rebars. The ESEM used was a Quanta FEG 250 from FEI (Hillsboro, OR, USA). The pictures were taken in secondary electron (SE) mode (low and high vacuum, voltage 5–15 kV, working distance 10 mm). Additionally, the roughness of the different surfaces was also quantified with a roughness meter (Mitutoyo Surftest SJ-210).

#### 2.3.2. Rebar Pullout Tests

**Figure 3:**
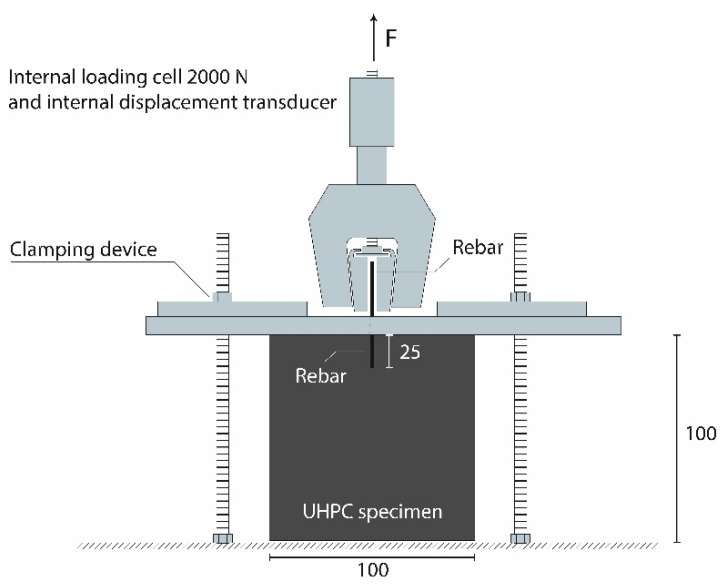
Setup for the rebar pullout tests. A universal testing machine (Zwick/Roell 150 kN) was used for the rebar pullout tests. An internal loading cell (2000 N) and a transducer were used to measure the pullout force and the pullout slip, respectively. Labelling in mm.

To quantify the bonding strength between the rebars and UHPC, rebar pullout tests were carried out using a Zwick/Roells 150 kN universal testing machine (Figure 3).

The rebar pullout tests were carried out in a displacement-controlled setup with a constant speed of 0.01 mm/s up to a pullout of 3 mm and a subsequent constant speed of 0.03 mm/s up to the complete pullout. This setup was used for all rebars. The samples had dimensions of 100 mm × 100 mm × 100 mm, and a single rebar was dipped centrally 25 mm (±2 mm) into the concrete from above and fixed to exclude sliding. Thus, the actual bonding length to calculate the bond stress was taken from the data gathered from the universal testing machine, i.e., the moment where no more force was obtained from the machine. Pullout force and displacement were determined by an internal loading cell (2000 N) and an internal displacement transducer.

With this setup, the bond stress of Fe-Mn-Al-Ni SMA rebars with diameters of 3 mm and 5 mm was determined. This was carried out for regular rebars, prestrained rebars, and prestrained and heated rebars. In the case of steel rebars, the tests were performed for regular as well as for roughened rebars, as the SEM investigations revealed a much smoother surface of the regular steel rebars (see Section 3.3). Roughening of the steel rebars was carried out manually with a steel brush perpendicularly to rebar pullout direction. This procedure increased the surface roughness of the steel rebars to an average roughness of 2.1 µm.

For labelling, the following system was applied (see also Table 2). The Fe-Mn-Al-Ni SMA rebars are labelled “FMAN”, while the steel rebars are referred to as “steel”. At the end, the state of the rebars is shown. In this regard, “p” stands for prestrained and “a” stands for activated rebars, while “Ref” indicates the reference, i.e., non-prestrained rebars with no thermal treatment. In the case of steel rebars, the “r” indicates a roughened surface. As an example, the results for the pullout test of a prestrained Fe-Mn-Al-Ni rebar with a diameter of 5 mm that is thermally activated afterwards are labelled as ‘FMAN5_ p+a’.

Using this data, the maximum bond stress ($\tau_{max}$) given in MPa is calculated by the following formula:

(1) $$\tau_{max}=\frac{F_{max}}{d_{r}\cdot \pi \cdot l_{e}}$$

where $F_{max}$ is the maximum pullout load, $d_{r}$ is the rebar diameter, and $l_{e}$ is the actual embedded rebar length [42].

The reached maximum rebar stress is calculated to quantify the efficiency of the different rebar types (utilisation factor $u_{f}$; see Equation (3)) [42]:

(2) $$\sigma_{r,max}=\frac{F_{max}}{A_{r}}=\frac{F_{max}}{\pi \cdot \frac{(d_{r}{)}^{2}}{4}}$$

In Equation (3) $u_{r}$ is defined as the ratio of the maximum tensile stress $\sigma_{max}$ of the rebar during pullout and the rebar’s tensile strength $r_{y}$. Here, a utilisation of more than 100% causes rebar rupture in the pullout process.

(3) $$u_{r}=\frac{\sigma_{r,max}}{r_{y}}\cdot 100$$

#### 2.3.3. Flexural Strength of Reinforced UHPC Prisms

**Figure 4:**
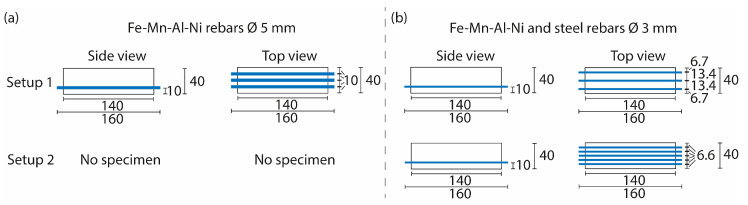
Setups of the rebars in the prisms for the flexural bending tests. (**a**) Setup for the Fe-Mn-Al-Ni rebars with a diameter of 5 mm. (**b**) Setups for the Fe-Mn-Al-Ni and steel rebars with a diameter of 3 mm. For both setups, Fe-Mn-Al-Ni as well as the steel rebars were used. Blue bars represent the rebars within the UHPC samples. Labelling in mm.

To investigate the influence of rebar material and the activation of the SMA rebars on the flexural strength of reinforced prisms and to compare these results to the rebar pullout tests, samples with two different setups were manufactured and tested (Figure 4).

**Figure 5:**
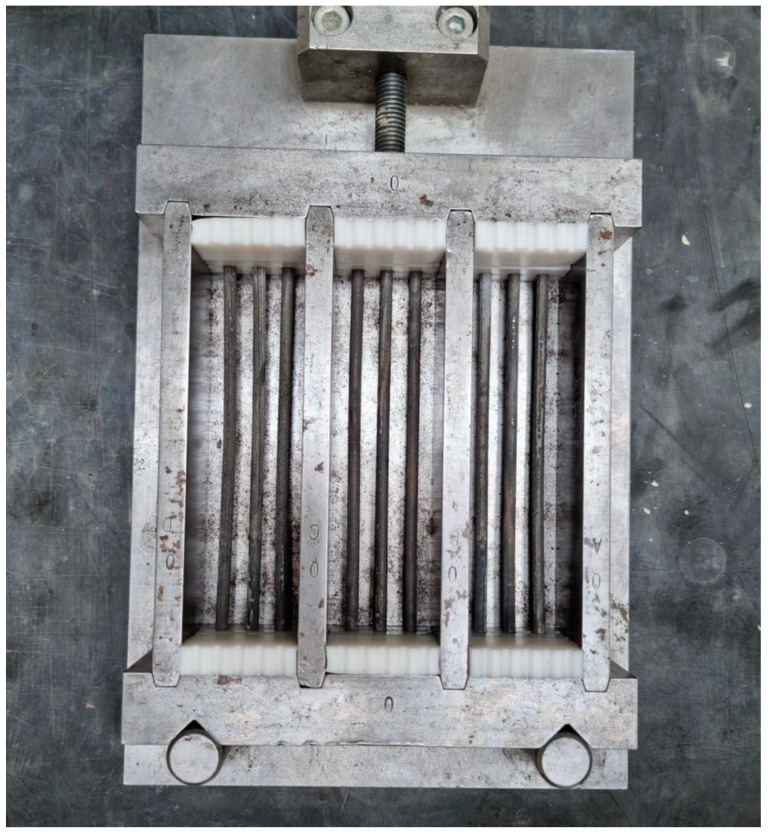
Image of a prism mould containing the fixation device and the fixed SMA rebars. In this example, the Ø 5 mm rebars in the setup 1 (see Figure 4) are shown. The regular moulds for the prisms have dimensions of 160 mm × 40 mm × 40 mm. Due to the 10 mm thick fixation devices, the dimensions are 140 mm × 40 mm × 40 mm.

For the SMA rebars with a diameter of 5 mm, only one setup with three rebars per UHPC sample was chosen (Figure 4, setup 1). In the case of the SMA rebars with a diameter of 3 mm and the steel rebars, two different setups were used, one of them with three rebars per UHPC sample (Figure 4, setup 1) and one with five rebars per UHPC sample (Figure 4, setup 2). For all specimens, the rebars were fixed at heights of 10 mm inside the UHPC prism. This was ensured using 3D-printed fixation devices. Due to these devices, the prisms had a length of 140 mm. Therefore, their dimensions were 140 mm × 40 mm × 40 mm (Figure 5). These dimensions are in accordance with the test setup in DIN EN 12390-5 [43].

The prisms gained by following setups were subjected to the activation heating process (see Section 2.1). To simplify the identification of the different combinations, the specimens were labelled based on the following system (see also Table 3). The Fe-Mn-Al-Ni SMA rebars are labelled “FMAN”, while the steel rebars are referred to as “steel”. The number after the material indicates the diameter of the used rebars. Afterwards, the setup type, e.g., “S1” for setup 1, is given. At the end, the state of the rebars is shown. In this regard, “p” stands for prestrained and “a” stands for activated rebars, while “Ref” indicates the reference, i.e., non-prestrained rebars with no thermal treatment. In the case of steel rebars, the “r” indicates a roughened surface. As an example, a UHPC prism reinforced with prestrained SMA rebars with a diameter of 3 mm placed in setup 2 and thermal activation afterwards is labelled as ‘FMAN3_S2_p+a’.

The flexural strength of the reinforced UHPC prisms was characterised using the same universal testing machine (Zwick/Roell 150 kN) based on a four-point bending tensile strength test. The tests were carried out at constant crosshead displacement speed of 0.01 mm/s according to DIN EN 12390-5 [43].

For quantitative characterisation of the maximum flexural strength and the post-failure behaviour, the average flexural strength at the point of deflection $\delta_{L1}=0.5\;\mathrm{mm}$ for the characteristic post-failure flexural strength of the service load range ($f_{cflk,L1}^{f}$) and $\delta_{L2}=3.5\;\mathrm{mm}$ in the fracture range ($f_{cflk,L2}^{f}$), as well as from the maximum load, were compared. This is based on the definition of the DAfStb guideline “Stahlfaserbeton” [44], which means that this evaluation is originally intended for fibre-reinforced concrete. However, as the prestress due to the SMA rebars will also have an influence on the flexural strength as well as on the post-failure behaviour, this method will also be used in this work. The average flexural strength at these characteristic points in N/mm^2^ is determined using the following equations:

(4) $$\;f_{cflm,L1}^{f}=\frac{1}{n}\sum_{i=1}^{n}\frac{M_{0.5i}}{W_{0.5i}}=\frac{1}{n}\sum_{i=1}^{n}\frac{\frac{F_{0.5i}}{2}\cdot \frac{l}{3}}{\frac{b_{i}\cdot h_{i}^{2}}{6}}=\frac{1}{n}\sum_{i=1}^{n}\frac{F_{0.5i}\cdot l}{b_{i}\cdot h_{i}^{2}}$$

(5) $$\;f_{cflm,L2}^{f}=\frac{1}{n}\sum_{i=1}^{n}\frac{F_{3.5i}\cdot l}{b_{i}\cdot h_{i}^{2}}$$

In Equations (4) and (5), F is the applied load [N], l is the support distance [mm], b is the section width [mm], h is the section height [mm], M is the moment [Nmm], W is the section modulus [mm^3^], and the index i is the sample number.

Due to the post-failure flexural strengths in the service and fracture load ranges, the performance characteristics of the fibre concretes are clearly describable. The characteristic values are required for the performance factor. These results depend on the coefficient of variation (*v*):

(6) $$\;\begin{array}{c} is\;v\leq 0.25:\;\;\;\;\;\;f_{cflk,Li}^{f}=0.51\cdot f_{cflm,Li}^{f} \end{array}$$

(7) $$\;\begin{array}{c} is\;v>0.25:\;\;\;\;\;f_{cflk,Li}^{f}=f_{cflm,Li}^{f}\cdot (1-t\cdot v) \end{array}$$

where t is the threshold for t distribution (5%—fractile).

This performance factor gives information about the material’s performance prior to failure (displacement of 0.5 mm) as well as the post-failure behaviour (displacement of 3.5 mm).

## 3. Results

### 3.1. Influence of Thermal Activation on the UHPC

To quantify the influence of the elevated temperatures on UHPC that occurs due to the thermal activation of the SMA rebars, the compressive and flexural strength of the UHPC were tested by treating non-reinforced UHPC specimens with the same heating procedure (see Section 2.1). The compressive and flexural strength of UHPC without any heat treatment are 122.5 MPa and 5.3 MPa, respectively. By heating the concrete up to 200 °C and holding this temperature for 2 h, the compressive and flexural strength increase to 200.0 MPa and 9.2 MPa, respectively.

### 3.2. Mechanical and Microstructural Properties of the Fe-Mn-Al-Ni SMA Rebars

The tensile strength of the Fe-Mn-Al-Ni SMA rebars is 770 ± 60 MPa, while the steel rebars have a tensile strength of 2065 ± 115 MPa. The chemical composition of the Fe-Mn-Al-Ni SMA rebars was characterised by energy-dispersive X-ray (EDX) spectroscopy (Table 4).

**Figure 6:**
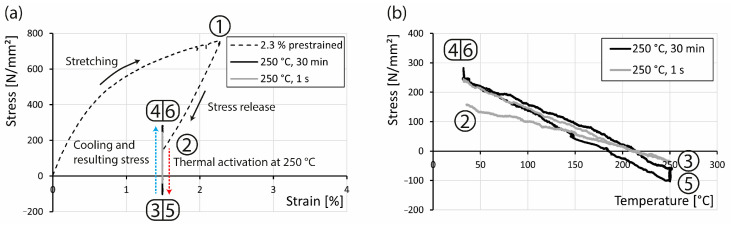
Prestraining process and the thermal activation of the Fe-Mn-Al-Ni SMA shown (**a**) in the stress/strain diagram and (**b**) in the stress/temperature diagram.

The expected prestress of the SMA rebars was tested in a tensile testing machine. The results show that the generated prestress is in the range of about 130 MPa (Figure 6). Therefore, the rebar was prestrained up to 2.3% (Point 1 in Figure 6a), and then the stress was released to 150 MPa to ensure that no buckling in the activation process would occur (Point 2 in Figure 6). The thermal activation was carried out in two steps. In the first one, the rebar was activated at 250 °C for 1 second (Point 3 in Figure 6), resulting in a loss of stress due to thermal expansion. This happens despite the activation of the shape memory effect. At the time of heating, the shape memory effect reduces the stress generated due to thermal expanse. Thus, in the following cooling of the rebar down to room temperature, a stress occurs due to thermal contraction (Point 4 in Figure 6), which can be attributed to the resulting prestress. To investigate the influence of the activation time, the rebar was heated again at 250 °C for 30 min (Point 5 in Figure 6). In the following cooling of the material, the stress increases again up to approx. 280 MPa, which results in a recovery stress of about 130 MPa (Point 6 in Figure 6). This stress level was achieved already at one second of heating. A longer heating period of 30 min leads only to a slight increase in generated stress.

### 3.3. Metallic and Surface Microstructure of the Fe-Mn-Al-Ni SMA Rebars

**Figure 7:**
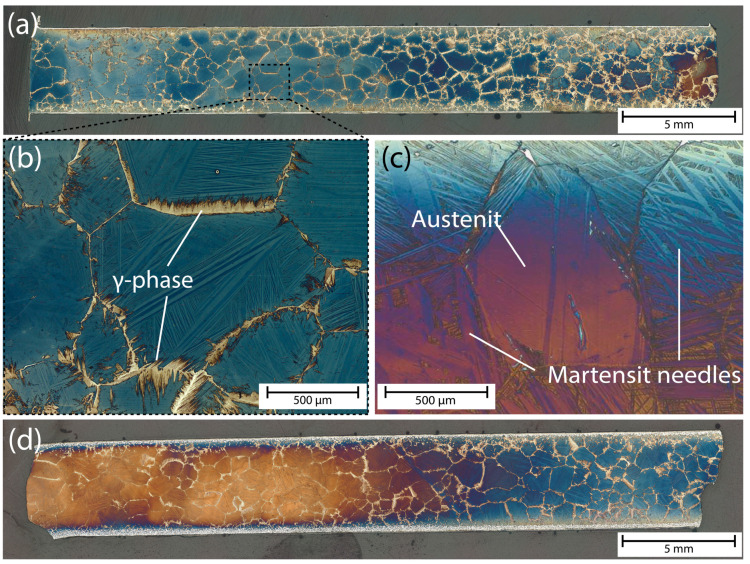
(**a**) Microstructural overview of a ~30 mm long section of a reference Fe-Mn-Al-Ni SMA rebar with a diameter of 5 mm. (**b**) Detailed view of a single metallic grain. Bright needle-like γ-phase at the grain boundaries is clearly abundant. (**c**) Detailed view showing austenitic phases within a single grain and surrounding martensite needles in another sample. (**d**) Microstructural overview of a ~30 mm long section of a 2% prestrained Fe-Mn-Al-Ni SMA rebar. Images were taken with a digital light microscope (Keyence VHX 7000). Colours are due to the Nital etching.

The metallic microstructure of the SMA rebars was recorded with a digital light microscope. To compare the different samples, overview shots with a magnification of 80× of all samples and more detailed shots of individual grains with a magnification of 150× were recorded. This was carried out for the reference samples (Figure 7a–c) and for the prestrained samples (Figure 7d).

The SMA rebars received solution heat treatment at 1250 °C. This austenitization temperature was chosen according to the phase diagram and data published in [45,46]. The rapid cooling rate (water quenching) after the solution heat treatment leads to a polycrystalline microstructure with an austenitic (α-phase) grain matrix, the formation of the non-reversible, ductile γ-phase on the grain boundaries (marked in Figure 7b), and scattered martensite plates within the grains (needle-like shapes within the grains marked in Figure 7c). Although the γ-phase is a non-transforming phase and therefore reduces the volume fraction of the phase showing a reversible phase transformation, the presence of this ductile phase on the grain boundaries is crucial for the investigated polycrystalline condition. Otherwise, the rapid cooling rate would lead to crack formation along the grain boundaries [47]. The formation of γ’-martensite prior to the prestraining process most likely results from the introduction of residual stresses during the quenching process. These stresses exceed the critical value for martensitic phase transformation, and a large fraction of martensite is formed [46,48]. However, the observed phase fraction of martensite within the grains is far larger than found in other studies for polycrystalline samples subjected to a similar heat treatment process [46], which might result from a slight variation in the chemical composition compared to other studies.

This high abundance of martensite counteracts the functionality of the rebar. This may have happened due to a slight shift in the materials’ chemical composition leading to different phase transformation temperatures or due to a flawed quenching procedure in the manufacturing process. Because high amounts of martensite are abundant prior to prestraining the rebars, only small amounts of stress-induced martensite, which is essential for the effectivity of the shape memory effect, can be formed by elongation of the rebars. An influence on the prestress capability of the material is not known but was also not investigated directly until now [35,47]. An additional heat treatment of the rebars did not lead to a more desirable microstructure.

Prestraining of the rebars shows no significant influence regarding the metallic microstructure. As high amounts of martensite were already existent prior to prestraining of the bars, whether and how much stress-induced martensite has formed due to the elongation process cannot be evaluated.

Because of these microstructural differences from a known Fe-Mn-Al-Ni SMA [35] as well as the generally low knowledge about this novel SMA, a distinct prognosis of the prestress capability of the rebars could not be determined prior to the experimental tests.

**Figure 8:**
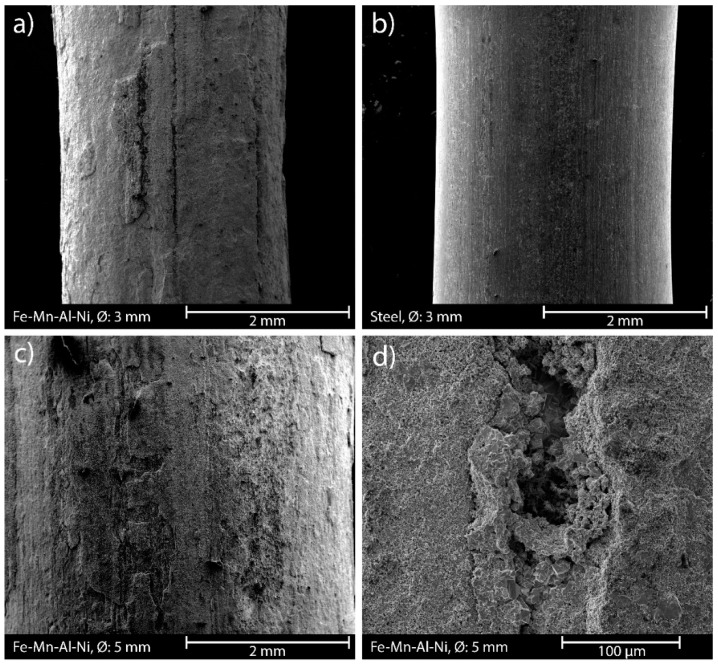
SEM images of the surfaces of the different rebars. (**a**) Surface of the Ø 3 mm SMA rebar; 35× magnification. (**b**) Surface of the Ø 3 mm steel rebar; 35× magnification. (**c**) Surface of the Ø 5 mm SMA rebar; 35× magnification. (**d**) Depression on the surface of the Ø 5 mm SMA rebar; 500× magnification. All images in SE mode in high vacuum.

Regarding the surface topography of the different rebar materials, significant differences were found by SEM and roughness meter investigation (Figure 8). The SMA rebars revealed a distinct topography on the surface (Figure 8d) with an average roughness of 3.3 µm. This is most likely due to corrosion of rebars. The same features are visible on all SMA rebars independent from their diameters (Figure 8a,c). In contrast, the steel rebars have a much smoother surface with an average roughness of 0.8 µm, but they also have some irregularities (Figure 8b).

### 3.4. Rebar Pullout Tests

The rebar pullout tests were carried out on SMA rebars and steel rebars to quantify the bond strength between the metallic surfaces and the cementitious UHPC matrix. This was carried out to show the influence of the rebar material, the rebar surface, and the process of prestraining and thermal activation of the SMA rebars on the bond.

**Figure 9:**
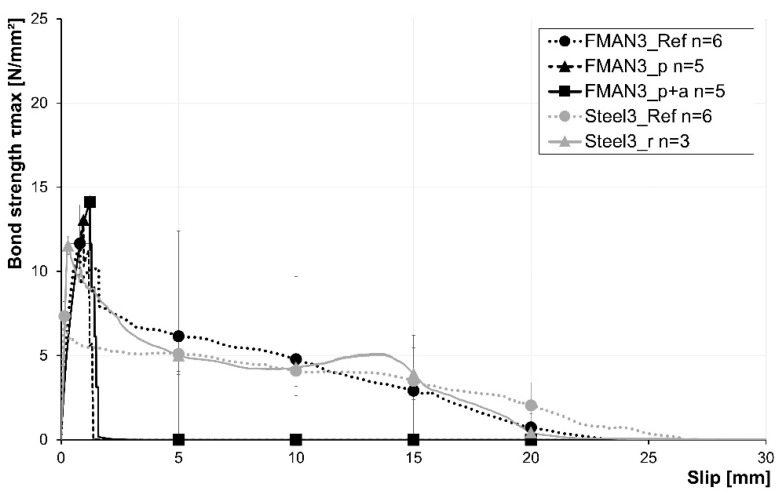
Results of the pullout tests for the rebars with a diameter of 3 mm. The black lines show the bond strength of the SMA rebars, and the grey lines show the bond strength for the steel rebars. Dotted, dashed, and solid lines indicate the different test setups corresponding to Section 2.3.1 Indicators for setups are not given, as only single rebar fibres were tested. n = number of samples.

Figure 9 shows the results of the rebar pullout tests of the SMA and steel rebars with a diameter of 3 mm. This includes the average stress–slip relationship of reference rebars for SMA and steel material as well as prestrained, prestrained, and annealed SMA and roughened steel rebars. Furthermore, the reached maximum bond stress, shown as bond strength, as well as the average bond stress at a slip of 5 mm, 10 mm, 15 mm, and 20 mm are given. The error indicators at these points show the respective standard deviations.

The results of the references for SMA (FMAN_Ref) and steel (Steel_Ref) reveal maximum bond stresses of 11.7 MPa and 7.4 MPa, respectively. It has to be mentioned that two of the six SMA reference specimens tore during the pullout test. Furthermore, the steel rebars reached their maximum pullout stress at a lower slip (0.15 mm) compared to the SMA rebars (0.81 mm). A roughening of the steel fibres increases the maximum bond stress about 55% to 11.5 MPa, which is in the range of the SMA reference rebars. Furthermore, the maximum pullout stress is reached at a higher slip of about 0.30 mm. However, this is still much lower compared to the SMA rebars. The post-failure behaviour after reaching the maximum bond stress is similar for the steel and FMAN_Ref specimens. The SMA reference showed a higher deviation at the five characteristic slip points compared to the steel samples.

In the case of the SMA rebars, the prestraining and the thermal treatment of the specimens led to a slight increase in the maximum bond strength. The prestraining led to a maximum pullout stress of 13.1 MPa, while an additional activation of the rebars led to a maximum pullout stress of 14.1 MPa. Moreover, the slip at maximum pullout stress also increased in the same manner from a slip of 0.81 mm (reference) to 1.23 mm (prestrained and activated rebars). All specimens of both rebar types tore during pullout, leading to the distinct stress drops during the test (Figure 9).

**Figure 10:**
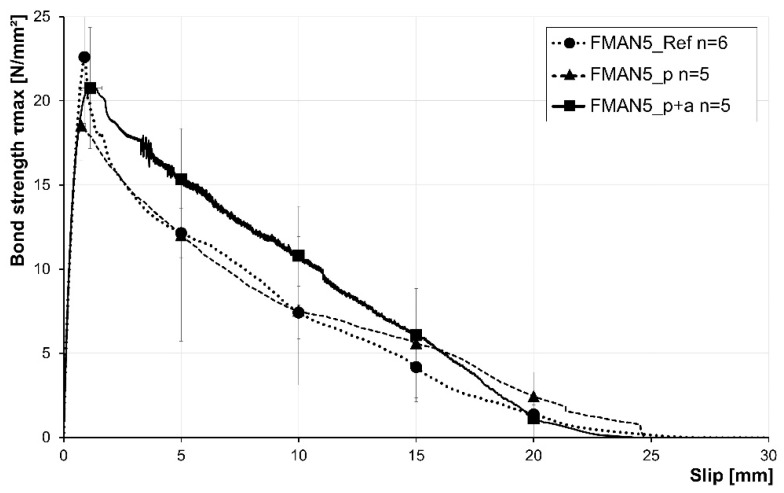
Results of the pullout tests for the SMA rebars with a diameter of 5 mm. Dotted, dashed, and solid lines indicate the different test setups corresponding to Section 2.3.2. Indicators for setups are not given, as only single rebar fibres were tested. n = number of samples.

In the case of the SMA rebars with a diameter of 5 mm, the improvement in bond strength due to the prestraining and activation of the rebars was not found (Figure 10). In contrast, prestraining of the rebars led to a decrease in maximum bond strength (18.6 MPa) compared to the reference (22.6 MPa), while prestraining and activation of the rebars also led to a slight decrease in bond strength (20.8 MPa) compared to the reference. However, an increase in the slip at maximum bond stress is also visible due to the prestraining and activation of the rebar. While the reference rebar reaches its maximum stress at a slip of about 0.88 mm, the prestrained and activated rebars reach their maximum bond stress at about 1.13 mm. The post-failure behaviour of the reference and the prestrained rebars is similar. In contrast, the prestrained and activated rebars led to higher pullout stresses until 15 mm and a subsequent alignment with the other samples. A summary of the rebar pullout test results is given in Table 5.

### 3.5. Flexural Strength

**Figure 11:**
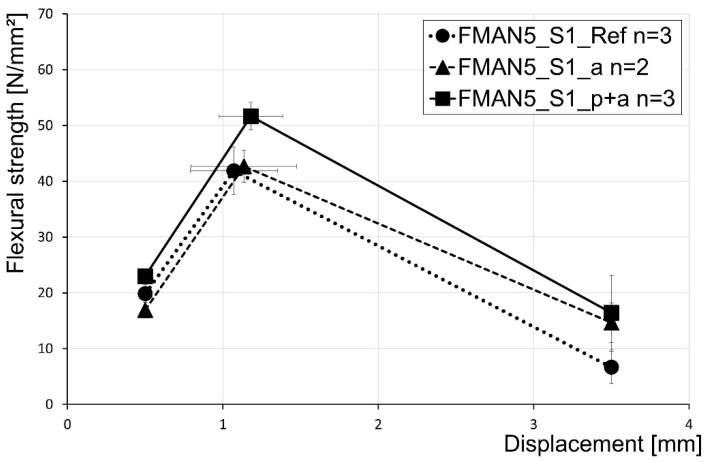
Results of the flexural strength tests of the SMA with a diameter of 5 mm characterised for three deflection points at 0.5 mm, 3.5 mm, and the displacement at maximum load.

The flexural strength of the reinforced UHPC prisms were determined for the different setups (material, dimensions, alignments) and heat treatment procedures (see Section 2.3.2). The SMA rebars with a diameter of 5 mm confirm the applied prestress due to the thermal activation of the rebars (Figure 11). In Figure 11, the flexural strength is shown for three characteristic displacement points of the test. Therefore, the flexural strengths at 0.5 mm displacement, 3.5 mm displacement, and the displacement of maximum load are given. The reference samples containing non-prestrained and non-heated rebars (FMAN5_S1_Ref in Figure 11) show a maximum flexural strength of 41.9 MPa. Beyond the fact that the thermal treatment itself led to the increase in the flexural strength of the UHPC due to thermal curing (see Section 3.1), for the next setup, non-prestrained SMA rebars were used, but the specimens were heated (FMAN5_S1_a in Figure 11). Thus, thermal curing of the UHPC was achieved, but no activation of rebars happened, as they were not prestrained. This did not lead to a significant increase in maximum flexural strength, which was 42.7 MPa. In contrast, the implementation of prestrained SMA rebars in UHPC and a subsequent heat treatment of those samples (FMAN5_S1_p+a in Figure 11) led to an increase of about 23% and 21% compared to the reference and the non-prestrained but also heated samples, respectively. A slight increase in the displacement at maximum flexural strength due to the activation of the rebars was also seen in these tests.

The characteristic deflection points at 0.5 mm and 3.5 mm revealed no significant differences, but there was a slight increase in flexural strength at 3.5 mm deflection ($f_{cflk,L2}^{f}$) from the reference (6.7 MPa) to the samples with prestrained and activated rebars (16.4 MPa). A summary of the flexural strength tests for UHPC prisms reinforced with SMA rebars with a diameter of 5 mm in setup 1 is given in Table 6.

**Figure 12:**
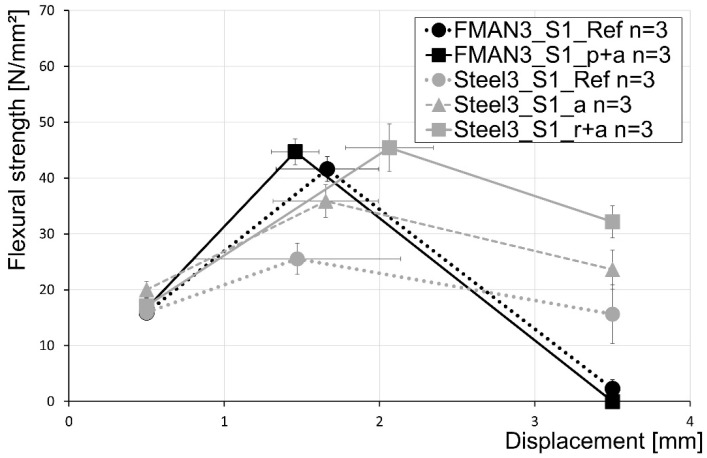
Results of the flexural strength tests for setup 1 of the SMA and steel rebars with a diameter of 3 mm characterised for three characteristic points at 0.5 mm displacement, 3.5 mm displacement, and the displacement at maximum load.

The results for the same setup (setup 1) but with rebars with a diameter of 3 mm are displayed in Figure 12. The results reveal that reinforcing of UHPC with SMA rebars led to a higher maximum flexural strength compared to the steel reference, with strengths of 41.6 MPa (FMAN3_S1_Ref in Figure 12) and 25.5 MPa (Steel3_S1_Ref in Figure 12), respectively. In contrast to the samples with SMA rebars, heating of the samples reinforced with steel rebars led to a significant increase in flexural strength (35.9 MPa; Steel3_S1_a in Figure 12). Additional roughening of the steel fibres increases the flexural strength further up to 45.4 MPa (Steel3_S1_r+a in Figure 12). This is even higher than that of the samples with activated SMA rebars, which have a flexural strength of 44.7 MPa (FMAN3_S1_p+a in Figure 12). Regardless, the activation of the SMA samples led to an increase in the flexural strength of 7.5% compared to the SMA reference samples.

Regarding the post-failure behaviour that is displayed in the $f_{cflk,L2}^{f}$-value, it is visible that the SMA rebars lead to significantly worse results compared to the steel rebars. In the case of the SMA reference, in one of the samples, all three rebars tore during testing, leading to no post-failure behaviour at all. This was also seen for all samples of the prestrained and activated SMA rebars with a diameter of 3 mm. Therefore, no post-failure behaviour can be described for the SMA samples, while the steel samples all show a decent post-failure behaviour, increasing from the reference to the roughened and thermally treated samples. A summary of the flexural strength tests for UHPC prisms reinforced with steel and SMA rebars with a diameter of 3 mm in setup 1 is given in Table 7.

**Figure 13:**
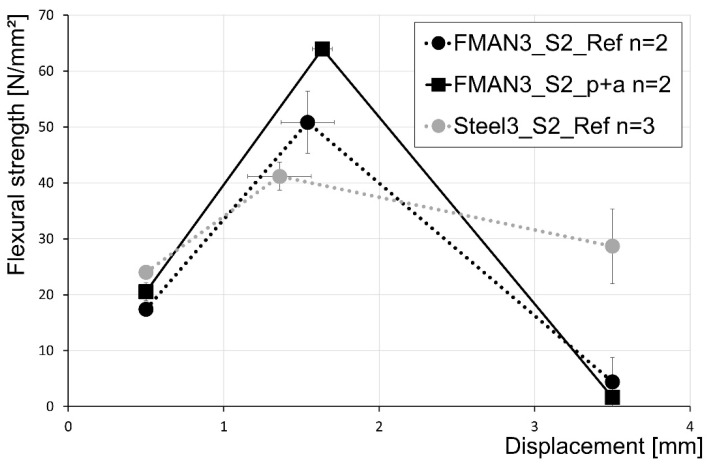
Results of the flexural strength tests for setup 2 of the SMA and steel rebars with a diameter of 3 mm characterised by three deflection points at 0.5 mm displacement, 3.5 mm displacement, and the displacement at maximum load.

More rebars (Setup 2) led to similar results; however, the prestress generated due to the activation of the SMA rebars is more significant (Figure 13). The reference of the SMA rebars (50.8 MPa; FMAN3_S2_Ref in Figure 13) led, again, to a higher flexural strength compared to the steel rebars (41.2 MPa; Steel_S2_Ref in Figure 13). Prior prestraining and thermal activation of the SMA rebars led to an increase of about 25.8% up to 64.0 MPa (FMAN3_S2_p+a in Figure 13) compared to the SMA reference. Furthermore, with increasing maximum flexural strength, the displacement at this point is also increased.

In the case of the $f_{cflk,L1}^{f}$-value, the steel rebars led to a slightly higher flexural strength compared to both SMA samples.

The values at 3.5 mm displacement ($f_{cflk,L2}^{f}$) revealed similar results as in setup 1. The steel samples retain a certain amount of their flexural strength up to this displacement, while the SMA samples tend to fail before the displacement, leading to an almost non-existent post-failure behaviour. A summary of the flexural strength tests for UHPC prisms reinforced with steel and SMA rebars with a diameter of 3 mm in setup 2 is given in Table 8.

## 4. Discussion

The focus of this study was on the capability of Fe-Mn-Al-Ni shape memory alloys to be used as tendons within UHPC. The aim was to generate a proof of concept for the general usability of the Fe-Mn-Al-Ni SMA as a prestressing tool in UHPC. This was investigated by quantifying the influence of the thermal activation of the prestrained SMA rebars and the generated prestress. Flexural bending tests of UHPC prisms reinforced with SMA and steel rebars in different test setups were carried out. Furthermore, rebar pullout tests were conducted to describe the influence of the activation process on the bond between rebar and cementitious matrix.

The microstructural investigations of the Fe-Mn-Al-Ni SMA revealed an unfavourable metallic microstructure for an application as a tendon. This resulted in a recovery stress that is significantly lower than regular prestressing steels but also is similar to iron-based shape memory alloys, which are often in range of about 250 MPa to 600 MPa [26,34,49], or the well-known NiTi-SMA, with recovery stresses of about 250 MPa to 500 MPa [50,51]. Thus, a quantitative comparison of the generated results is not directly suitable, but a proof of concept for the use of Fe-Mn-Al-Ni shape memory alloys as a prestressing element in UHPC was achieved.

The flexural bending tests revealed the prestress potential of the novel Fe-Mn-Al-Ni SMA that was proposed by Vollmer et al. [35]. In all setups, prestraining and thermal activation of the samples led to an increase in maximum flexural strength compared to the untreated SMA rebars. This is supported by the fact that heating of samples with unprestrained SMA rebars did not lead to a significant increase in maximum flexural strength. Therefore, this increase can be attributed directly to the activation of the shape memory effect within the prestrained SMA rebars. However, this effect was mainly visible in the samples containing rebars with a diameter of 5 mm. The samples with the 3 mm rebar reinforcement did not show a significant improvement by thermal activation of the rebars in either tested setup. Furthermore, heating of the samples reinforced with 3 mm steel rebars, revealed a significant increase in maximum flexural strength. This is supposed to be due to the generally lower bond strength between steel and the UHPC matrix. The heating shows a more prominent influence on the bond strength compared to the SMA rebars. In the case of the SMA rebars, a sufficient bond is gained by the rough surface (Figure 8), which superimposes the effects of heating on bond strength. Due to the low-volume fraction of reinforcement in these samples, the shape memory effect is not that distinct and did not lead to a significant improvement regarding maximum flexural strength.

A direct quantitative comparison with the steel rebars is not suitable, as the surface structure and the mechanical properties of the materials are significantly different. As both characteristics have distinct influence on the bond strength [12,40,52] and, therefore, on the performance as reinforcement, the results regarding steel rebars have to be interpreted in a more qualitative than quantitative manner. The SMA rebars with a diameter of 3 mm led to a similar flexural strength after prestraining and thermal activation compared to the roughened and thermally treated steel rebar samples. Due to relatively low maximum tensile strength combined with the good bond due to the rough surface of the SMA material, most of the rebars tore in the testing process. The failure could also be promoted by the microstructure of the rebars, as the surfaces also revealed cracks and depressions (see Figure 8). More cracks inside the rebars, therefore, cannot be excluded. These defects lead to no existent post-failure behaviour for the samples with SMA reinforcement, and, thus, a quantitative comparison with the steel rebars is not suitable. However, the samples with 5 mm SMA rebars reveal a worse post-failure behaviour compared to the 3 mm steel rebars. This is in accordance with the rebar pullout tests, as the steel rebars led to a slightly better post-failure behaviour compared to the SMA rebars. Thus, a direct relationship of pullout behaviour and post-failure behaviour cannot be assumed, which is also shown in literature [12,53]. To achieve a better comparability with the steel samples, the Fe-Mn-Al-Ni alloy must be designed with similar mechanical, microstructural, and topographic characteristics. This would counteract tearing of the rebars during the tests and should allow much higher resulting flexural strengths of the reinforced concrete. Possible ways to achieve this could include the addition of a fifth element into the alloy system as well as different quenching procedures in the manufacturing process, as these parameters have a significant influence on the mechanical and microstructural characteristics of the material [54].

Sawaguchi et al. investigated the prestress potential of Fe-Mn-Si SMA [55]. They reinforced prisms (20 mm × 20 mm × 80 mm) made of a high-strength mortar with prestrained SMA wires with a diameter of 2 mm. A recovery stress of about 250 MPa was determined for these wires. By activating the wires, the flexural strength of the mortar was increased in a range of about 15% to 40%, depending on the activation temperature and duration. Shahverdi et al. revealed an even higher increase in flexural strength using Fe-Mn-Si-Cr-Ni SMAs as reinforcement bars inside a normal-strength concrete (compressive strength: 53 MPa) [56]. The rebars were placed inside grooves that were cut out of the concrete and were afterwards filled with a cement-based grout. Prior to testing, the rebars with a recovery stress of 220 MPa to 250 MPa were thermally activated. The activation led to an increase in maximum bearing load of about 80% in relation to the non-activated samples. Compared to the results of this study, where a maximum increase in flexural strength was about 26%, the more distinct influence could be assigned to the higher recovery stresses of the used reinforcement rebars. This is supported by the fact that Vollmer et al. revealed generated prestresses of 485 MPa with the same alloy system that was used in this study [35]. The comparably low prestress in this study can be assigned to the high abundances of martensite in the initial state of the rebars. Therefore, by limiting the martensite percentage in this state, higher prestress levels can be assumed [35].

Kromoser et al. examined the feasibility of an iron-based SMA-grid as prestressing reinforcement in UHPC [23]. The reinforcement in the form of a grid has the advantage of a much better bond between the SMA and the matrix, as the cross braces of the grid serve as anchorage inside the UHPC. Therefore, they tested reference and reinforced UHPC beams. The activation of the SMA led only to a slight increase in the bending strength of the beams compared to the non-activated samples. They found that the activation led to an increase in the flexural rigidity of the beams, as the samples with the activated SMA revealed a much higher displacement at maximum force compared to the non-activated samples. This is in accordance with the results of this study, as the displacement at maximum flexural strength also increases mostly with the activation of the SMA rebars.

The rebar pullout tests revealed a much higher bond strength of the SMA rebars compared to the steel rebars. This was expected due to the much rougher SMA surface (see Section 3.3) due to corrosion products [12,52]. This difference in bond strength was eliminated by roughening of the steel rebars. Compared to NiTi-SMA, the bond strength is significantly higher for the Fe-Mn-Al-Ni SMA [40,57]. Other studies mentioned a lower bond strength of a Fe-Mn-Si-Cr-Ni SMA rebar compared to regular steel rebars, which contrasts with the results of this study [58].

The prestraining of the SMA rebars and the heating of the specimens had a slight negative influence on the bond stress, but not in a significant manner. This is in accordance with the literature, where it is stated that activation temperatures of ≤200 °C only led to slight changes in bond strength [59,60]. Regarding the results in this study, the slight decrease could be due to the expansion of the rebars during activation. When the rebars are prestrained, the diameter is reduced slightly. When retaining its original shape due to thermal activation within the UHPC matrix, the SMA rebars transfer a stress onto the surrounding concrete, analogical to the Hoyer effect [61]. When these stresses are too large, cracking around the rebar could occur, resulting in loss of bond strength. However, as regular prestressing steels mostly have a rippled surface structure, this could also be applied onto SMA rebars, and, therefore, those would have a much more prominent impact on the bond compared to the differences regarding rebar material [10].

Due to the low tensile strength of the SMA rebars, some samples tore during testing. This applies to the rebar pullout tests as well as to the flexural bending tests. The latter is especially unfavourable, as no post-failure behaviour could be achieved due to rupture of the rebars. This was mainly found in the setups using the thinner rebars. In these setups, the steel rebars led to better performance factors, as much higher stresses could be received after failure of the UHPC matrix compared to the SMA rebars. Furthermore, due to the lower tensile strength of the SMA rebars, the maximum tensile strength achieved is just about in the range of the roughened steel rebars. Therefore, to achieve even better values compared to regular steel rebars, the alloy has to be adjusted in terms of tensile strength, as a higher tensile strength of the reinforcement increases the mechanical properties of the component [10].

In general, the ability of Fe-Mn-Al-Ni SMA to be used as prestressing tendon in UHPC was shown within this work. As a next step, the SMA has to be adjusted in order to improve its mechanical properties and also to improve the stability of the system, i.e., to ensure the necessary microstructure. Therefore, the manufacturing process has to be further investigated, and the influence of the processing routes (annealing temperature and time, quenching temperature and time, etc.) on the microstructure and the transformation temperatures has to be characterised [35,62,63,64]. Another aspect resulting from the processing route is the chemical composition of the alloy. The alloy composition of the used material differs from known and functional samples [35] by up to 13 at.% in regards to the four main elements of the Fe-Mn-Al-Ni SMA. This is, of course, related to the composition of the base material prior to the manufacturing of the rebars; however, the annealing and quenching processes can also lead to differences in chemical composition and microstructure [35,65,66]. As the rebars used in this work were annealed at 1250 °C together with Mn pellets within steel foil, the Mn pellets could have led to a slightly higher Mn concentration in the SMA samples. Mn is known to be a stabilizer for the γ-phase [47]. That could explain the high amounts of γ-phase in the SMA samples of this study.

In terms of application in civil engineering, different aspects have to be investigated in further research. As mentioned before, the influence of stronger rebars has to be examined. With stronger rebars, a stronger bond can also be used to create even higher prestresses. Furthermore, long-term investigations must be carried out. One of the most beneficial properties of the Fe-Mn-Al-Ni SMA is that they can hold the prestress over long strains [35,67,68,69]. Therefore, this ability could counteract prestress losses due to shrinkage and creep of the concrete, which is a problem for existing prestressing concepts [70,71]. A combination of fibre and SMA rebar reinforcement could also be used in order to increase the effectiveness of the shape memory effect. A stronger bond between rebar and UHPC due to fibre reinforcement could lead to a more effective implementation of the prestress [72]. Alignment of the fibres could improve this effect even further [5]. However, this improvement in bond strength must be in accordance with the tensile strength of the used SMA rebars.

These aspects will be investigated in future research. The proposed method reveals a huge potential, and a proof of concept is shown. The used Fe-Mn-Al-Ni alloy has unique characteristics that allow novel applications and will counteract known issues of regular prestressing systems. These novel applications must be investigated further, and the results shown in this study are the basis for upcoming research. However, quantitative comparison with other methods is not applicable here, as too many open questions are still existent.

## 5. Conclusions and Outlook

The use of Fe-Mn-Al-Ni SMA rebars as prestress tendons in UHPC was investigated and discussed in this study. To reveal the influence of rebar material and surface as well as different reinforcement setups, rebar pullout tests and four-point bending tests of UHPC prisms reinforced with SMA and steel rebars were carried out. Based on the results, the following conclusions can be drawn:

- The prestressing capability of Fe-Mn-Al-Ni SMA rebars as reinforcement in UHPC was clearly shown in this work. Prestraining and thermal activation of the SMA rebars with a diameter of 5 mm within the UHPC matrix led to a significant improvement in the maximum flexural strength as well as flexural rigidity of the UHPC specimen.
- A sufficient amount of SMA reinforcement is necessary to have a significant influence of the shape memory effect. Samples reinforced with 3 mm SMA rebars only showed slight increases in maximum flexural strength due to the thermal activation. The samples containing steel rebars revealed an increase in maximum flexural strength by thermal treatment that could superimpose the shape memory effect.
- The SMA rebars have a rougher surface compared to steel rebars. This is mainly due to corrosion processes in manufacturing and storage. The rough surface leads to high bond strengths between metallic rebar and the cementitious UHPC matrix. However, as the SMA rebars have a lower tensile strength, the good bond leads to partial rupture of the rebars and, therefore, to a bad or non-existing post-failure behaviour.
- The manufacturing route of the SMA rebars needs to be optimised and investigated further to ensure the mechanical and functional properties of the SMA. This includes annealing and quenching conditions of the rebars to ensure an optimal microstructure. The microstructure of the material as well as its chemical composition strongly influence the functionality of the material. Higher maximum tensile strength and a more effective shape memory effect of the rebars will increase the resulting flexural strength of the concrete even further. However, there are also other aspects of the rebars that have to be considered, e.g., yield strength, Young’s modulus of elasticity, and Poisson´s ratio [10,73].
- The activation process for the reinforced UHPC specimen must be optimised. As temperatures of about 200 °C can be problematic to be applied in precast fabrication, the alloy has to be designed with lower activation temperatures. These should be in the range of regular curing temperatures of precast concrete elements, i.e., in a range of about 60 °C to 80 °C [74]. This allows a more applicable method of SMA activation.

## Figures and Tables

**Table 1:** Composition of the UHPC mixture.

Compounds	kg/m^3^	wt. %
CEM I 52.5 R HS/NA	797	34.0
Silica fume: Sika Silicoll P	169	7.2
Quartz sand: G32 0.125 mm/0.5 mm	966	41.2
Quartz powder: Millisil W12	199	8.5
Superplasticizer: Sika Viskocrete 2810	27	1.1 bwoc *
Water	187	8.0
w/b-ratio **	0.21	

* bwoc: by weight of cement; ** w/b: water/binder ratio.

**Table 2:** Labelling system for the rebar pullout test specimens.

Material	Diameter [mm]	State	Abbreviation
Fe-Mn-Al-Ni	3	reference	FMAN3_Ref
		prestrained	FMAN3_p
		prestrained and thermally activated	FMAN3_p+a
	5	reference	FMAN5_Ref
		prestrained	FMAN5_p
		prestrained and thermally activated	FMAN5_p+a
Steel	3	reference	Steel3_Ref
		roughened	Steel3_Ref

**Table 3:** Labelling system for the flexural strength test specimens.

Material	Diameter [mm]	Setup	State	Abbreviation
Fe-Mn-Al-Ni	3	Setup 1	reference	FMAN3_S1_Ref
			prestrained and thermally activated	FMAN3_S1_p+a
		Setup 2	reference	FMAN3_S2_Ref
			prestrained and thermally activated	FMAN3_S2_p+a
	5	Setup 1	reference	FMAN5_S1_Ref
			thermally activated	FMAN5_S1_a
			prestrained and thermally activated	FMAN5_S1_p+a
Steel	3	Setup 1	reference	Steel3_S1_Ref
			thermally treated	Steel3_S1_a
			roughened and thermally treated	Steel_S1_r+a
		Setup 2	reference	Steel3_S2_Ref

**Table 4:** Proportions of Fe, Mn, Al, and Ni in the used SMA, determined by EDX spectroscopy.

Element	at. %
iron	40.2 (±0.02)
manganese	37.3 (±0.22)
aluminium	14.9 (±0.29)
nickel	7.6 (±0.09)

**Table 5:** Results of the rebar pullout tests for the rebars with a diameter of 3 mm; matrix UHPC was used.

Characteristic	F_max_	r_y_	d_r_	sF_max_	τ_max_	σ_r,max_	u_f_
Unit	kN	MPa	mm	mm	MPa	MPa	%
FMAN3_Ref	4.22 (±0.8)	770	3.0	0.81	11.7 (±2.3)	597	77.5
FMAN3_p	5.53 (±0.1)	770	3.0	0.96	13.1 (±0.1)	782	101.6
FMAN3_p+a	5.60 (±0.1)	770	3.0	1.23	14.1 (±0.4)	792	102.9
FMAN5_Ref	7.34 (±0.4)	770	5.0	0.88	22.6 (±2.1)	374	48.6
FMAN5_p	6.79 (±0.8)	770	5.0	0.75	18.6 (±4.0)	346	44.9
FMAN5_p+a	7.41 (±1.5)	770	5.0	1.13	20.8 (±3.6)	377	49.0
Steel_Ref	1.71 (±0.2)	2065	3.0	0.15	7.4 (±0.9)	242	11.7
Steel_r	2.47 (±0.3)	2065	3.0	0.30	11.5 (±0.6)	349	16.9

F_max_: maximum pullout load. r_y_: tensile strength of the rebar. sF_max_: rebar pullout load at maximum pullout load. d_r_: rebar diameter. τ_max_: maximum bond stress. σ_r,max_: maximum reached rebar tensile stress. u_f_: capacity utilization of the rebar. W_p_: pullout work.

**Table 6:** Comparison of the flexural strength values of SMA rebars with a diameter of 5 mm.

	FMAN5_S1_Ref			FMAN5_S1_a			FMAN5_S1_p+a		
	δ_L1_	δ_L2_	δ_L(max)_	δ_L1_	δ_L2_	δ_L(max)_	δ_L1_	δ_L2_	δ_L(max)_
Mean forces [kN]	12.70	4.26	26.80	10.83	9.38	27.30	14.73	10.50	33.06
Standard deviation F [kN]	0.34	0.91	2.76	1.31	4.23	2.38	0.29	0.96	2.79
Standard deviation S [mm]	0.00	0.00	0.05	0.00	0.00	0.05	0.00	0.00	0.12
Average f_cflm_ [MPa]	19.85	6.66	41.88	16.91	14.66	42.66	23.01	16.41	51.65
f_cflm_ [MPa]	10.12	3.40	21.36	8.63	1.84	21.75	11.74	8.37	26.34
Performance factor	L 10.12/3.40			L 8.88/1.84			L 11.74/8.37		

**Table 7:** Comparison of the flexural strength values of steel and SMA rebars with a diameter of 3 mm in setup 1.

	**Steel3_S1_Ref**				**Steel3_S1_a**				**Steel3_S1_r+a**			
	**δ_L1_**	**δ_L2_**		**δ_L(max)_**	**δ_L1_**	**δ_L2_**		**δ_L(max)_**	**δ_L1_**	**δ_L2_**		**δ_L(max)_**
Mean forces [kN]	10.20	10.00		16.35	12.80	15.12		22.95	10.94	20.59		29.07
Standard deviation F [kN]	0.99	4.10		2.18	1.13	2.77		2.28	1.24	2.27		3.31
Standard deviation S [mm]	0.00	0.00		0.67	0.00	0.00		0.34	0.00	0.00		0.28
Average f_cflm_ [MPa]	15.94	15.63		25.54	20.01	23.63		35.85	17.09	32.17		45.42
f_cflm_ [MPa]	5.20	2.03		8.34	10.20	12.05		18.29	8.72	16.41		23.17
Performance factor	L 5.20/2.03				L 10.20/12.05				L 8.72/16.41			
	**FMAN3_S1_Ref**						**FMAN3_S1_p+a**					
	**δ_L1_**		**δ_L2_**		**δ_L(max)_**		**δ_L1_**		**δ_L2_**		**δ_L(max)_**	
Mean forces [kN]	10.18		1.46		26.62		10.68		0.00		28.61	
Standard deviation F [kN]	0.64		1.26		1.74		1.43		0.00		1.79	
Standard deviation S [mm]	0.00		0.00		0.33		0.00		0.00		0.15	
Average f_cflm_ [MPa]	15.91		2.29		41.59		16.69		0.00		44.70	
f_cflm_ [MPa]	8.11		0.00		8.47		8.51		0.00		22.78	
Performance factor	L 4.71/0.00 *						L 8.51/0.00					

* Because all rebars tore during the test in one of the samples, the ratio from standard deviation to mean force became too large, and a negative performance factor would be the result. Because of this, the performance factor was set to 0.00 here.

**Table 8:** Comparison of the flexural strength values of steel and SMA rebars with a diameter of 3 mm in setup 2.

	Steel3_S2_Ref			FMAN3_S2_Ref			FMAN3_S2_p+a		
	δ_L1_	δ_L2_	δ_L(max)_	δ_L1_	δ_L2_	δ_L(max)_	δ_L1_	δ_L2_	δ_L(max)_
Mean forces [kN]	15.38	18.35	26.35	11.15	2.83	32.54	13.16	1.07	40.93
Standard deviation F [kN]	0.98	5.22	1.96	0.67	4.00	5.05	1.45	1.51	0.55
Standard deviation S [mm]	0.00	0.00	0.20	0.00	0.00	0.17	0.00	0.00	0.06
Average f_cflm_ [MPa]	24.03	28.68	41.17	17.41	4.41	50.84	20.55	1.67	63.95
f_cflm_ [MPa]	12.25	13.08	21.00	8.88	0.00 *	25.93	10.48	0.00 *	32.62
Performance factor	L 12.25/13.08			L 8.88/0.00 *			L 10.48/0.00 *		

* Because all rebars tore during the test in one of the samples, the ratio from standard deviation to mean force became too large, and a negative performance factor would be the result. Because of this, the performance factor was set to 0.00 here.

## Data Availability

Data is unavailable due to privacy restrictions.
